# A Clinico-Genotypic Prognostic Index for *De Novo* Composite Diffuse Large B-Cell Lymphoma Arising from Follicular Lymphoma in Asian patients treated in the Rituximab Era

**DOI:** 10.1038/s41598-020-61378-4

**Published:** 2020-03-09

**Authors:** Ryan Mao Heng Lim, Natalie Pei Xin Chan, Lay Poh Khoo, Chee Leong Cheng, Leonard Tan, Eileen Yi Ling Poon, Nagavalli Somasundaram, Mohamad Farid, Tiffany Pooi Ling Tang, Miriam Tao, Soon Thye Lim, Jason Yongsheng Chan

**Affiliations:** 1 Yong Loo Lin School of Medicine, National University of Singapore, Singapore, Singapore; 2 Division of Medical Oncology, National Cancer Centre Singapore, Singapore, Singapore; 3 Department of Anatomical Pathology, Singapore General Hospital, Singapore, Singapore; 4 SingHealth Duke-NUS Blood Cancer Centre, Singapore, Singapore; 5 Duke-NUS Medical School, Singapore, Singapore; 6 Cancer Science Institute of Singapore, National University of Singapore, Singapore, Singapore

**Keywords:** Tumour biomarkers, Non-hodgkin lymphoma, Cancer genomics

## Abstract

Composite follicular lymphoma with diffuse large B-cell lymphoma (FL/DLBCL) is uncommonly found on lymph node biopsy and represents a rare haematological malignancy. We aim to examine clinico-pathological features of patients with FL/DLBCL and investigate predictors of survival outcome. We included in our retrospective study patients with histologically-proven FL/DLBCL at diagnosis (n = 106) and who were subsequently treated with rituximab-based chemoimmunotherapy from 2002–2017 at the National Cancer Centre. The cohort consisted of 34 women and 72 men with a median age of 59 years (range, 24–82). In a multivariate model inclusive of known clinico-pathological parameters at diagnosis, advanced stage (*p* = 0.0136), presence of *MYC* and/or *BCL6* rearrangement (*p* = 0.0376) and presence of B symptoms (*p* = 0.0405) were independently prognostic for worse overall survival (OS). The only remaining independent prognostic variables for worse OS after including first-line treatment data in the model were use of chemotherapy regimens other than R-CHOP (*p* = 0.0360) and lack of complete response to chemotherapy (*p* < 0.0001) besides the presence of B symptoms (*p* = 0.0022). We generated a Clinico-Genotypic Index by point-wise addition of all five adverse parameters (score of 0–1, 2, 3, 4–5) which revealed four prognostic risk groups with a predicted 5-year OS of 100%, 62%, 40% and 0% (*p* < 0.0001) accounting for 50.0%, 24.5%, 18.9% and 6.6% of the cohort respectively. We propose that R-CHOP should be the recommended first-line regimen for composite FL/DLBCL.

## Introduction

Follicular lymphoma (FL) is among the most common subtype of non-Hodgkin lymphoma (NHL), accounting for over one-fifth of NHL worldwide^1^. It is a heterogeneous group of tumours that originate from centrocytes and centroblasts among germinal centre B cells^2^. It is also well-known that FL has a propensity to transform to diffuse large B-cell lymphoma (DLBCL) at a rate of approximately 3% per year for the first 15 years^3,4^. Histological transformation (HT) to DLBCL portends a poor prognosis with a median survival of 14 to 27 months^3,5,6^.

Multiple studies have elucidated various clinical and molecular markers predicting for HT and overall survival (OS) post-HT. High-risk Follicular Lymphoma International Prognostic Index (FLIPI), grade 3 histology, stage III or IV disease, low serum albumin and high serum lactate dehydrogenase (LDH) levels are commonly cited as risk factors for HT^5,7–9^. Molecular alterations such as *TP53*^10^ and *CDKN2A/B* mutations^11^, as well as rearrangements of *BCL6*^12^ and *MYC*^13^ have also been implicated in HT. High-risk FLIPI, advanced age, poor performance status, and elevated serum β2-microglobulin levels were found to be associated with poor OS post-HT^6^.

Most of the current literature describes the behavior and prognosis of low grade FL and its subsequent HT. However, in clinical practice, lymph node biopsy often yields composite or discordant histology of both FL and DLBCL (FL/DLBCL). One recent study suggested that synchronous FL/DLBCL at diagnosis denotes outcomes intermediate between FL and DLBCL (5-year OS: 85%, 73% and 63% for FL, FL/DLBCL and DLBCL respectively)^14^, while another study suggests that the prognosis of concurrent FL/DLBCL is similar to that of germinal centre B-cell-like (GCB)-subtype DLBCL^15^. Few studies have investigated the behavior, prognostic factors and optimal management of biopsy-proven synchronous FL/DLBCL at the point of histological diagnosis. In addition, relevant data in these aspects gathered in the rituximab era has been lacking, despite contemporary treatment recommendations commonly including rituximab-based chemoimmunotherapy without upfront autologous stem-cell transplantation. Finally, it is also not well understood whether a FL/DLBCL at diagnosis represents a distinct lymphoma entity or the process of HT from FL to DLBCL.

Hence in this study, we examine the clinical outcomes of FL/DLBCL treated in the rituximab era and investigate the prognostic utility of various clinical, pathological, and molecular biomarkers. In doing so, we propose a concise scoring system to aid clinicians to better prognosticate and manage this unique entity.

## Patients and Methods

### Study cohort

Patients who were consecutively diagnosed with synchronous composite or discordant follicular lymphoma (FL) and diffuse large B-cell lymphoma (DLBCL) (defined henceforth as FL/DLBCL) and seen at the National Cancer Centre Singapore and Singapore General Hospital between November 2002 and January 2017 were retrospectively analysed. A total of 106 patients who were treated with a rituximab-based chemotherapy regimen were included in the final analysis. The 106 patients were chosen from a patient pool of 1529 DLBCL cases and 428 FL cases. Patient with composite FL/DLBCL comprised 5.4% of patients diagnosed with either FL or DLBCL. The median follow-up duration for this group of patients was 48.0 months.

Relevant demographical and clinicopathological information were collected and utilised for the analysis. Demographical information available included sex, age and ethnicity. Clinical characteristics of each patient including B-symptoms, Eastern Cooperative Oncology Group (ECOG) performance status, chemotherapeutic regimen and response to treatment were included in the study. Finally, pathological information such as tumour grade, tumour stage, number of lymph nodes involvement, number of extranodal site involvement, various immunohistochemical markers (BCL2, BCL6, CD10, MUM1, KI-67, MYC), gene rearrangements (*BCL2*, *BCL6*, *MYC*) in the DLBCL component, and DLBCL subtype by Han’s algorithm^16^ were included in the analysis.

Two expert haematopathologists (C.L.C. and L.T.) reviewed all patient biopsies. Cases of diagnostic difficulty were discussed in detail at a histo-morphology meeting and tumour board before a consensus diagnosis was made to reduce the rate of discordant diagnosis. In the diagnosis of FL, apart from follicular dendritic meshworks by CD21 and/or CD23, the overall architecture, as well as the presence of centrocytes with centroblasts, were thoroughly assessed. The FL component was graded as per WHO criteria as Grade 1: 0–5 centroblasts/high-power field (HPF), Grade 2: 6–15 centroblasts/HPF, and Grade 3: > 15 centroblasts/HPF (3A if centrocytes are present and 3B if centrocytes are absent). The DLBCL component was defined as an area of large cells in sheets lacking follicular architecture assessed by staining for follicular dendritic cells (CD21 or CD23), and is distinct from grade 3B FL where present^17^. The percentage of DLBCL was estimated based on relative proportion to the entire specimen examined.

Informed consent for the use of biospecimens was obtained in accordance with the Declaration of Helsinki and all methods were carried out in accordance with relevant guidelines and regulations. All data was obtained at the time of diagnosis or subsequent follow-up. The research study was carried out with approval from the SingHealth Centralised Institutional Review Board. Participants and/or their legal guardians provided informed consent for their data to be used in this research. The datasets created and analysed during this study are available from the corresponding authors upon reasonable request.

### Statistical analysis

The outcomes of interest in this study are overall survival (OS) and progression-free survival (PFS). OS was calculated from the date of diagnosis up to the date of death from any cause, or was censored at the date of last follow-up for survivors. PFS was defined as the time elapsed between the date of diagnosis to the date of relapse, progression, or death from any cause. For each individual clinicopathological parameter, Kaplan-Meier survival curves were plotted to estimate survival. The log-rank test was then used to determine hazard ratio (HR), the corresponding 95% confidence intervals of mortality and the p-values for each individual clinicopathological characteristic. Clinicopathological parameters found to be significant on univariate analysis using a two-sided test with significance level of 0.05 were identified. Subsequently, parameters with significance level of <0.10 were used in the generation of Multivariate Cox regression models via a stepwise procedure to test for independence of significant factors at diagnosis and after collection of first-line treatment data. Independently significant variables were tested for association with response to first-line chemotherapy with the chi-squared test. Statistical analysis was carried out using methods as previously described^18^.

A prognostic scoring model excluding first-line treatment data was created with each independently significant variable at the point of diagnosis attributed a point, and a Kaplan-Meier survival curve plotted to compare survival between patients scoring 0, 1, and 2–3 on the index. Incorporating treatment data and outcomes, a Clinico-Genotypic Index was created with each independently significant variable attributed a point, and a Kaplan-Meier survival curve was plotted to compare survival between patients scoring 0–1, 2, 3, and 4–5 points on the index. All tests were performed using MedCalc statistical Software for Windows version 19.0.4 (MedCalc Software, Ostend, Belgium).

## Results

### Patient demographics and clinicopathological characteristics

The median age of diagnosis was 59 years (range: 24–82 years). Seventy-two (67.9%) were male and 34 (32.1%) were female. All 106 patients were diagnosed with histologically-proven synchronous FL/DLBCL, with the range of DLBCL component for all patients between 10–100%. Clinical and demographic data are summarised in Table 1 while pathological characteristics of the patients are summarised in Table 2. The DLBCL component was assessed to be <50% in 29 (27.4%), ≥ 50% in 69 (65.1%), and unknown in 8 (7.5%) patients. Twenty-eight (26.4%) had grade 1–2 FL while 78 (73.6%) had grade 3 FL at diagnosis. Forty-two (39.6%) had an Ann Arbor stage of 1–2 while 64 (60.4%) were diagnosed as stage 3–4. 100 patients had bone marrow biopsy at diagnosis, with 27 (27.0%) of them being positive for lymphomatous involvement. 5 out of the 27 patients with lymphomatous involvement of the bone marrow had discordant low grade FL, with DLBCL histology at the primary biopsy site. The majority of patients achieved objective responses to chemotherapy (81.1%), with 67.0% achieving complete response (CR). The R-CHOP regimen (rituximab, cyclophosphamide, doxorubicin hydrochloride, vincristine, prednisolone) was most frequently prescribed (84.0%). 5 patients received Rituximab maintenance therapy while no patients received upfront autologous stem cell transplant.

**Table 1:** Clinical and demographic characteristics.

Characteristic	N (%)
Total	106 (100%)
***Age (years)***	
> 60	50 (47.2%)
< 60	56 (52.8%)
***Sex***	
Male	72 (67.9%)
Female	34 (32.1%)
***Ethnicity***	
Chinese	71 (67.0%)
Malay	13 (12.3%)
Indian	8 (7.5%)
Others	14 (13.2%)
***B-symptoms***	
Absent	74 (69.8%)
Present	32 (30.2%)
***ECOG performance status***	
0	62 (58.5%)
1–4	44 (41.5%)
***Stage***	
1–2	42 (39.6%)
3–4	64 (60.4%)
***FLIPI***	
0–1	35 (33.0%)
2	23 (21.7%)
3	48 (45.3%)
***IPI***	
0–1	39 (36.8%)
2	29 (27.3%)
3	26 (24.5%)
4–5	12 (11.3%)
***Response to chemotherapy***	
Complete response (CR)	71 (67.0%)
Non-CR	35 (33.0%)
***First line chemotherapy regimen***	
R-CHOP	89 (84.0%)
Others*	17 (16.0%)

Abbreviations: FLIPI, Follicular Lymphoma International Prognostic Index; IPI, International Prognostic Index.

*Other regimens include RCVP (n = 4), R-EPOCH (n = 3), RICE (n = 2), CHOP (n = 1), Rituximab + Cyclophosphamide (n = 1), RCOP (n = 1), RECP (n = 1), RCEOP (n = 1), RGDP (n = 1), RCP (n = 1), RCEPP (n = 1).

**Table 2:** Histopathological and molecular characteristics.

Characteristic*	N
***Grade***	
1–2	28 (26.4%)
3	78 (73.6%)
3A	64 (60.4%)
3B	9 (8.5%)
Unknown	5 (4.7%)
***Number of lymph node involvement***	
1–3	81 (76.4%)
≥ 4	25 (23.6%)
***Number of extra-nodal involvement***	
0	46 (43.4%)
1–2	50 (47.2%)
3–7	9 (42.3%)
***Bone Marrow Involvement***	
Negative	73 (73.0%)
Positive	27 (27.0%)
***Serum LDH***	
Not elevated	35 (33.0%)
Elevated	71 (67.0%)
***DLBCL component***	
< 50%	29 (27.4%)
≥ 50%	69 (65.1%)
Unknown	8 (7.5%)
***BCL2 expression***	
Negative	24 (24.2%)
Positive	75 (75.8%)
***BCL6 expression***	
Negative	6 (7.0%)
Positive	80 (93.0%)
***CD10 expression***	
Negative	32 (30.8%)
Positive	72 (69.2%)
***MUM1 expression***	
Negative	24 (28.2%)
Positive	61 (71.8%)
***Ki-67 expression***	
< 90%	68 (72.3%)
> 90%	26 (27.7%)
***MYC expression***	
< 40%	41 (70.7%)
> 40%	17 (29.3%)
***Cell of origin***	
ABC	62 (60.8%)
GCB	40 (39.2%)
***BCL2 rearrangement***	
Negative/unknown	87 (82.1%)
Positive	19 (17.9%)
***BCL6 rearrangement***	
Negative/unknown	90 (84.9%)
Positive	16 (15.1%)
***MYC rearrangement***	
Negative/unknown	101 (95.3%)
Positive	5 (4.7%)
***BCL6*** **and/or** ***MYC*** **rearrangement**	
Negative/unknown	85 (80.2%)
Positive	21 (19.8%)
**Double-hit**	
Negative/unknown	103 (97.2%)
Positive	3 (2.8%)

Abbreviations: LDH, lactate dehydrogenase; DLBCL, diffuse large B-cell lymphoma; ABC, activated B-cell-like; GCB, germinal centre B-cell-like.

*Parameters with unknown data include bone marrow involvement (n = 6), immunohistochemical expression for BCL2 (n = 17), BCL6 (n = 20), CD10 (n = 2), MUM1 (n = 21), Ki-67 (n = 12) and MYC (n = 48), as well as genomic rearrangement for *BCL2* (n = 55), *BCL6* (n = 53), and *MYC* (n = 56).

### Molecular pathological characteristics

Immunohistochemical analysis showed the following rates of positive expression for each marker: BCL2–75/99 (75.8%); BCL6–80/86 (93.0%); CD10–72/104 (69.2%); MUM-1–61/85 (71.8%); Ki-67 index ≥ 90% – 26/94 (27.7%), MYC staining ≥ 40% – 17/58 (29.3%). Fluorescence *in situ* hybridisation (FISH) revealed genetic rearrangements at the following rates: *BCL2*–19/106 (17.9%), *BCL6*–16/106 (15.1%), *MYC* – 5/106 (4.7%). Three out of 106 patients (2.8%) were double-hit (rearrangements of *MYC*, *BCL2* and/or *BCL6*) while 21/106 (19.8%) had *MYC* and/or *BCL6* rearrangements.

### Survival analyses

**Figure 1:**
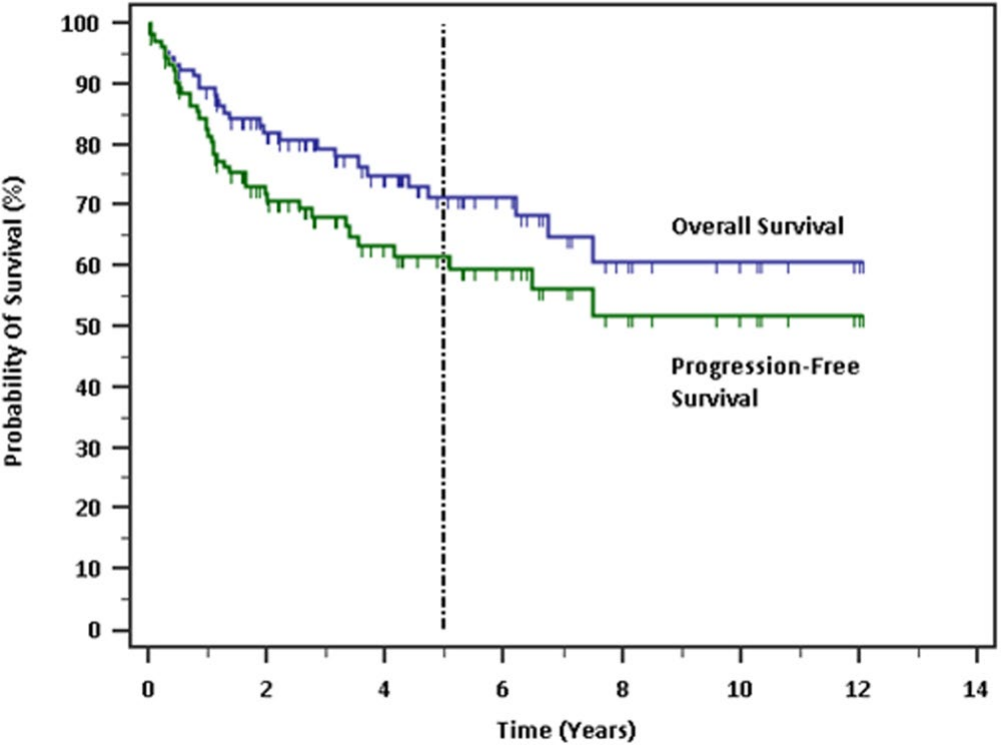
Survival probabilities of the global cohort.

**Figure 2:**
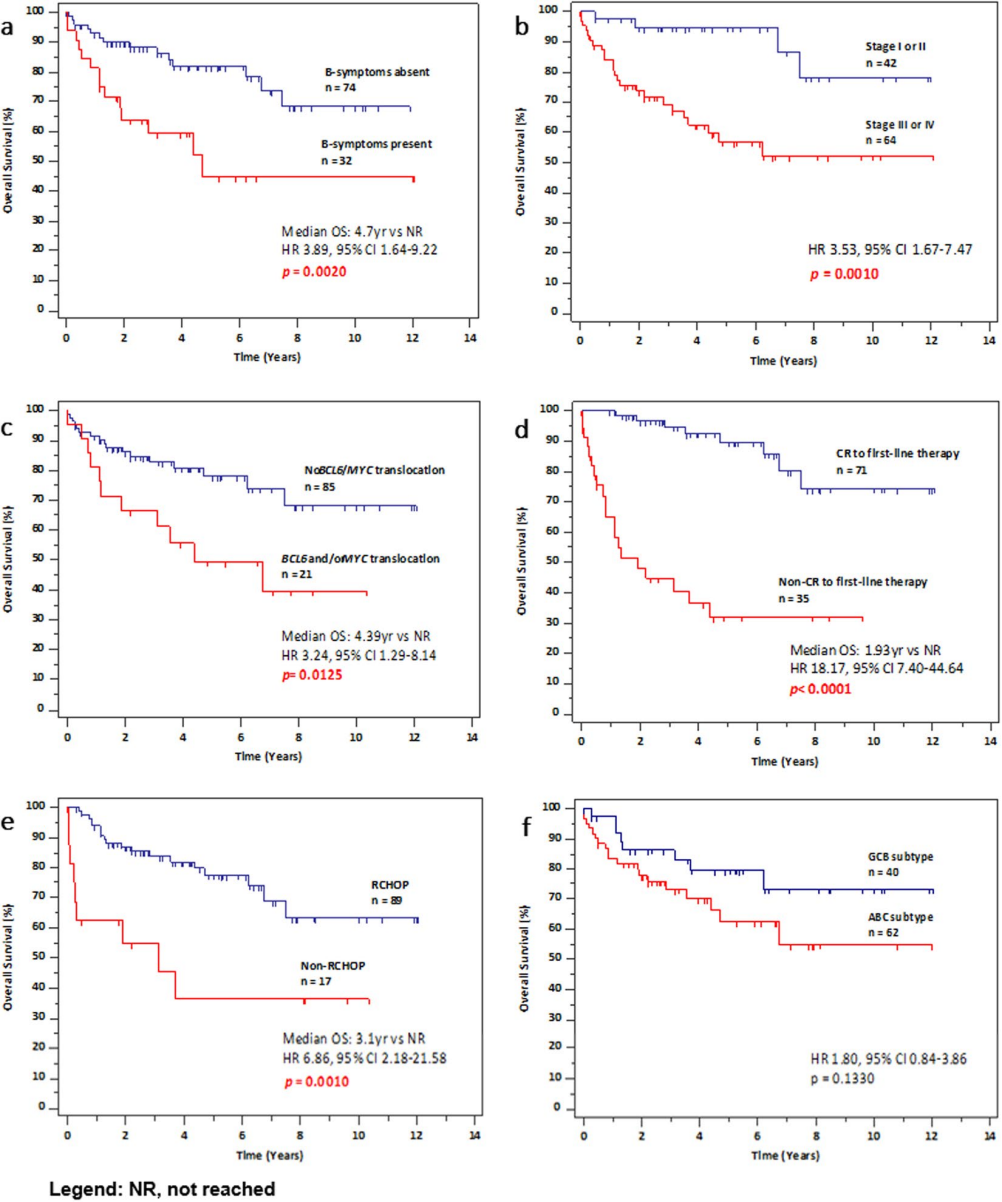
Kaplan-Meier survival analysis. (**a**) Presence of B-symptoms, (**b**) stage III or IV, (**c**) *BCL6* and/or *MYC* rearrangement, (**d**) non-CR to first-line chemotherapy, (**e**) use of chemotherapy regimen other than R-CHOP were predictive of worse overall survival. (**f**) Poorer OS was observed for the ABC subtype as compared to the GCB subtype, though this was not statistically significant (p = 0.1330).

At the time of data analysis, 24 patients (22.6%) had died. The 5-year OS and PFS of the global series is 71.2% and 61.6% respectively (Fig. 1). In univariate analysis, presence of B-symptoms at diagnosis (HR 3.89, 95% CI 1.64–9.22, p = 0.0020), stage 3–4 (HR 3.53, 95% CI 1.67–7.47, p = 0.0010), involvement of 4 or more lymph nodes at diagnosis (HR 2.75, 95% CI 1.08–7.00, p = 0.0337), presence of *BCL6* and/or *MYC* rearrangements (HR 3.24, 95% CI 1.29–8.14, p = 0.0125), non-CR to first-line chemotherapy (HR 18.17, 95% CI 7.40–44.64, p < 0.0001), and use of chemotherapy regimen other than R-CHOP (HR 6.86, 95% CI 2.18–21.58, p = 0.0010) were significantly correlated with worse OS (Fig. 2 and Table 3). The subgroup of grade 3B FL (n = 9) trended towards worse OS (HR 3.36, 95% CI 0.88–12.78, p = 0.0757) and PFS (HR 2.78, 95% CI 0.87–9.00, p = 0.0842) over grade 1–3A.

**Table 3:** Clinicopathological factors investigated for overall survival and progression-free survival.

Characteristic	Overall survival			Progression-free survival		
	HR	95% CI	*p*-value	HR	95% CI	*p*-value
B-symptoms (Present vs Absent)	3.89	1.64–9.22	**0.0020**	1.90	0.92–3.90	0.0808
ECOG performance status (1–4 vs 0)	1.80	0.85–3.82	0.1234	1.83	0.96–3.50	0.0671
Stage (3–4 vs 1–2)	3.53	1.67–7.47	**0.0010**	2.66	1.40–5.06	**0.0028**
Grade (3 vs 1–2)	0.48	0.20–1.12	0.0890	0.51	0.24–1.07	0.0744
No. of lymph node involvement (≥4 vs 1–3)	2.75	1.08–7.00	**0.0337**	3.21	1.42–7.24	**0.0050**
Response to chemotherapy (Non-CR vs CR)	18.17	7.40–44.64	**<0.0001**	10.88	4.89–24.23	**<0.0001**
Chemotherapy regimen (Others vs R-CHOP)	6.86	2.18–21.58	**0.0010**	2.98	1.12–7.94	**0.0287**
BCL2 expression (Positive vs Negative)	1.29	0.55–3.02	0.5529	1.81	0.87–3.76	0.1146
BCL6 expression (Positive vs Negative)	0.69	0.13–3.63	0.6584	0.55	0.13–2.38	0.4275
CD10 expression (Positive vs Negative)	0.83	0.36–1.90	0.6537	1.05	0.51–2.17	0.8916
MUM1 expression (Positive vs Negative)	1.76	0.75–4.13	0.1958	1.86	0.88–3.96	0.1061
KI-67 expression (≥90% vs < 90%)	1.06	0.41–2.77	0.9053	0.59	0.28–1.27	0.1801
MYC expression (≥40% vs < 40%)	0.63	0.20–1.96	0.4267	0.98	0.34–2.83	0.9757
Cell of origin (ABC vs GCB)	1.80	0.84–3.86	0.1330	1.71	0.88–3.32	0.1105
*BCL2* rearrangement (Positive vs Negative/unknown)	2.04	0.78–5.36	0.1488	2.18	0.91–5.22	0.0788
*BCL6* rearrangement (Positive vs Negative/unknown)	2.60	0.94–7.17	0.0653	2.00	0.82–4.90	0.1275
*MYC* rearrangement (Positive vs Negative/unknown)	4.37	0.72–26.40	0.1077	2.18	0.47–10.05	0.3193
*BCL6* and/or *MYC* rearrangement (Negative/unknown)	3.24	1.29–8.14	**0.0125**	2.18	0.97–4.87	0.0581

Abbreviations: ECOG, Eastern Cooperative Oncology Group.

In terms of PFS, stage 3–4 (HR 2.66, 95% CI 1.40–5.06, p = 0.0028), involvement of 4 or more lymph nodes at diagnosis (HR 3.21, 95% CI 1.42–7.24, p = 0.050), non-CR to chemotherapy (HR 10.88, 95% CI 4.89–24.23, p < 0.0001), and use of chemotherapy regimen other than R-CHOP (HR 2.98, 95% CI 1.12–7.94, p = 0.0287) were predictive of poorer outcomes (Table 3). Classification by cell of origin by Han’s algorithm did not reach statistical significance as a predictor for OS. However, patients with the activated B-cell-like (ABC) subtype tended to have a shorter OS as compared to those with the germinal centre B-cell-like (GCB) subtype (p = 0.1330) (Fig. 2).

We proceeded to create a multivariate model adjusted for significant clinicopathological parameters for OS and PFS at two crucial junctures. First, Cox’s multivariate proportional hazard analysis was performed inclusive only of all known significant clinical and pathological parameters at the point of diagnosis. They are: presence of B-symptoms, grade 3, stage 3–4, involvement of 4 or more lymph nodes, *BCL6* rearrangement, and *BCL6* and/or *MYC* rearrangement. Here, presence of B-symptoms at diagnosis (HR 2.24, 95% CI 1.04–4.83, p = 0.0405), stage 3–4 (HR 3.94, 95% CI 1.33–11.69, p = 0.0136), and presence of *BCL6* and/or *MYC* rearrangement (HR 2.24, 95% CI 1.05–4.80, p = 0.0376) were all independently associated with poorer OS. Only stage 3–4 (HR 3.11, 95% CI 1.42–6.79, p = 0.0045) was independently associated with poorer PFS (Table 4).

**Table 4:** Cox’s multivariate analysis for significant clinicopathological factors excluding treatment outcomes.

Parameter	Overall survival			Progression-free survival		
	HR	95% CI	*p*-value	HR	95% CI	*p*-value
***B-Symptoms***						
Absent	1	1.04–4.83	**0.0405**	—		
Present	2.24					
***Stage***						
1–2	1	1.33–11.69	**0.0136**	1	1.42–6.79	**0.0045**
3–4	3.94			3.11		
***BCL6 and/or MYC rearrangement***						
Absent	1	1.05–4.80	**0.0376**	—		
Present	2.24					

Next, multivariate analysis was performed for all significant clinicopathological parameters after incorporating first-line treatment data. They include all the above parameters as well as non-CR response to first-line chemotherapy and use of other chemotherapy regimen from R-CHOP. Presence of B-symptoms at diagnosis (HR 3.50, 95% CI 1.57–7.81, p = 0.0022), non-CR response to first-line chemotherapy (HR 7.78, 95% CI 3.29–18.41, p < 0.0001) and use of other chemotherapy regimen from R-CHOP (HR 2.46, 95% CI 1.06–5.71, p = 0.0360) were all independently associated with poorer OS. Only non-CR response to first-line chemotherapy (HR 5.74, 95% CI 2.97–11.07, p < 0.0001) was independently prognostic for poorer PFS (Table 5).

**Table 5:** Cox’s multivariate analysis for significant clinicopathological factors including treatment outcomes.

Parameter	Overall survival			Progression-free survival		
	HR	95% CI	*p*-value	HR	95% CI	*p*-value
***B-symptoms***						
Absent	1	1.57–7.81	**0.0022**			
Present	3.50					
***Response to chemotherapy***						
CR	1	3.29–18.41	**<0.0001**	1	2.97–11.07	**<0.0001**
Non-CR	7.78			5.74		
***Chemotherapy regimen***						
R-CHOP	1	1.06–5.71	**0.0360**			
Others	2.46					

### Clinico-genotypic index

**Figure 3:**
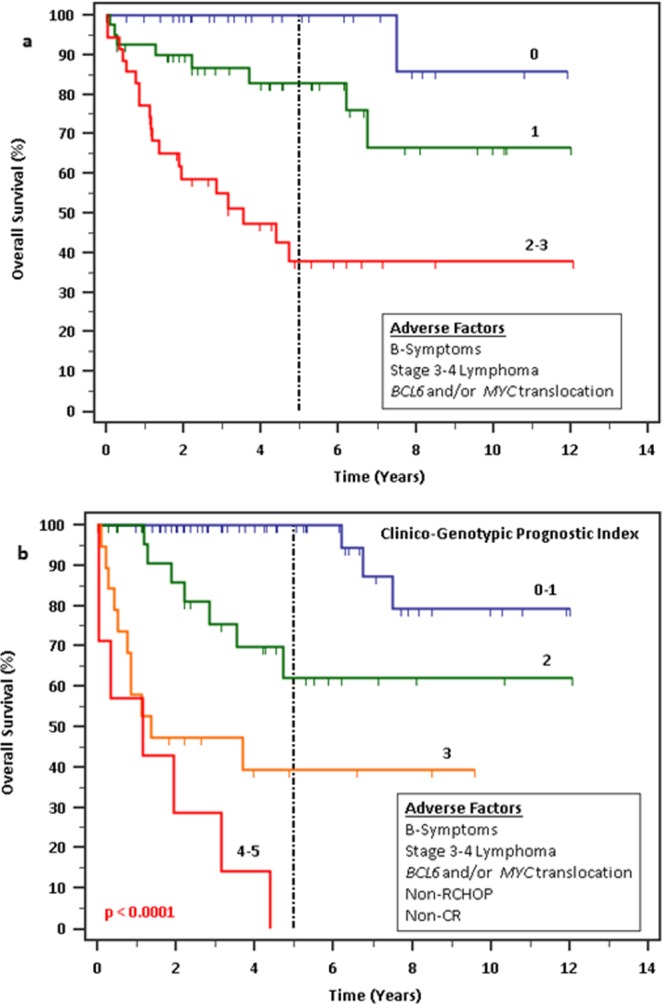
Survival outcomes by prognostic scoring. (**a**) Prognostic scoring without inclusion of first-line treatment data, revealing 3 groups of patients derived from point-wise addition of each adverse factor for OS. (**b**) Overall survival outcomes by prognostic scoring using Clinico-Genotypic Index (CGI), revealing 4 groups of patients derived from point-wise addition of each independent adverse factor with incorporation of treatment data. Higher number of points on both scoring indices significantly predicted for poorer OS (p < 0.001).

An initial prognostic model was derived from point-wise addition of the 3 adverse parameters for OS before inclusion of first-line treatment data with score classifications of 0 (low risk), 1 (intermediate risk) and 2–3 (high risk). This revealed 3 prognostic risk groups for patients accounting for 28.3%, 38.7% and 33.0% of the cohort, with a predicted 5-year OS of 100%, 83% and 38% (p < 0.0001) and a predicted 5-year PFS of 89%, 67% and 36% respectively (p = 0.0007) (Table 6) (Fig. 3a). Higher risk scores (p = 0.0037) and use of chemotherapy regimens apart from R-CHOP (p = 0.0003) were associated with non-CR to first-line chemotherapy.

**Table 6:** Prognostication based on adverse parameters excluding first-line treatment data.

Parameter	HR	95% CI	5-year OS/PFS	*p*-value
***Overall survival***				
0 Point	1	—	**100%**	<0.0001
1 Point	6.38	2.66–15.31	**83%**	
2–3 Points	22.16	8.47–57.99	**38%**	
***Progression-free survival***				
0 Point	1	—	**89%**	0.0007
1 Point	1.98	0.93–4.21	**67%**	
2–3 Points	4.74	2.09–10.72	**36%**

We were able to better predict the survival outcomes of patients by including post-treatment clinical and genotypic data. A Clinico-Genotypic Index (CGI) was derived from point-wise addition of all 5 unique adverse parameters from the 2 multivariate analyses above (presence of B-symptoms at diagnosis, stage 3–4 lymphoma, presence of *BCL6* and/or *MYC* rearrangement, non-CR response to first-line chemotherapy and use of other chemotherapy regimen from R-CHOP) with score classifications of 0–1 (low risk), 2 (low-intermediate risk), 3 (high-intermediate risk) and 4–5 (high risk). This revealed 4 prognostic risk groups for patients accounting for 50.0%, 24.5%, 18.9% and 6.6% of the cohort, with a predicted 5-year OS of 100%, 62%, 40% and 0% and a predicted 5-year PFS of 81%, 60%, 41% and 0% respectively (p < 0.0001) (Table 7) (Fig. 3b).

**Table 7:** Prognostication based on points scored on Clinico-Genotypic Index.

Parameter	HR	95% CI	5-year OS/PFS	*p*-value
***Overall survival***				
0–1 Point	1	—	**100%**	<0.0001
2 Points	5.66	2.31–13.82	**62%**	
3 Points	16.15	5.26–49.62	**40%**	
4–5 Points	39.30	5.11–302.04	**0%**	
***Progression-free survival***				
0–1 Point	1	—	**81%**	<0.0001
2 Points	1.67	0.78–3.57	**60%**	
3 Points	4.67	1.79–12.20	**41%**	
4–5 Points	11.16	1.85–67.44	**0%**	

**Figure 4:**
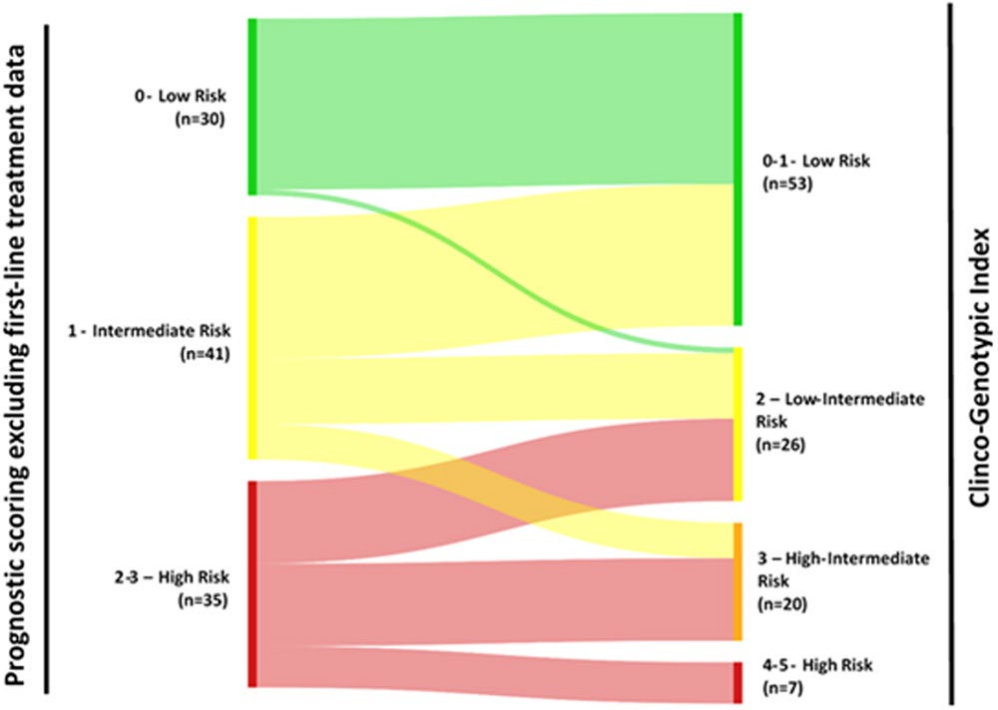
Classification of patients into initial prognostic scoring and final CGI.

Using the CGI, we were able to more precisely stratify patients into 4 unique prognostic risk groups as compared to the 3 risk groups using the earlier initial prognostic model. Most patients initially categorised as low risk remained in the same group, with 3.3% reclassified into low-intermediate risk. Intermediate risk patients were reclassified into low (58.5%), low-intermediate (26.8%) or high-intermediate (14.6%) risk groups. High risk patients were reclassified into low-intermediate (40.0%), high-intermediate (40.0%) and high (20.0%) risk groups (Fig. 4). Patients in the high risk group had the poorest median overall survival of only 13.4 months.

### Analysis at disease relapse

From the patient cohort, 23 patients had experienced disease relapse during the period of follow-up. The median time to relapse is 16.2 months. 12 patients relapsed as DLBCL, 7 patients relapsed as FL, 3 relapsed as composite FL/DLBCL, while the histology for 1 patient was unknown.

### Comparison between synchronous and metachronous FL and DLBCL

We compared the clinicopathological characteristics of 106 cases of synchronous (composite) FL/DLBCL with that of 21 cases of metachronous FL/DLBCL (FL that had undergone subsequent HT to DLBCL). Comparison between clinical and demographical characteristics are summarised in Supplementary Table 1 while comparison between histopathological and molecular characteristics are summarised in Supplementary Table 2. Comparing the prognosis of synchronous FL/DLBCL against metachronous FL/DLBCL at the point of transformation, the latter had a significantly worse OS (HR 4.29, 95% CI 1.58–11.65, p = 0.0042) (Supplementary Fig. 1).

## Discussion

Our current study demonstrates that a concise Clinic-Genotypic Index can classify patients with composite FL and DLBCL histology at diagnosis into distinct prognostic groups. We did not observe any significant prognostic associations with various immunohistochemical markers (BCL6, CD10, MUM1, Ki-67 and MYC), as well as classification by cell of origin using Han’s algorithm. However, we showed that patients with FL/DLBCL positive for *BCL6* and/or *MYC* rearrangements carried a poorer prognosis as compared to those who did not have either alteration.

In previous studies done with patients receiving first-line treatment with R-CHOP, an important prognostic factor for *de novo* DLBCL has been found to be translocation of *MYC*^19–22^ in addition to the well-established International Prognostic Index (IPI) for DLBCL^23^. Two studies conducted in the rituximab era also reported an association of *BCL6* translocation with poorer OS^24,25^. In our study, while *MYC* translocation does not reach statistical significance, there is a clear trend towards poorer OS and PFS. This may be a result of the relatively small sample size. Incorporating data on *BCL6* rearrangement, *MYC* and/or *BCL6* translocation were shown to be independent variables for poor OS. Taken together, composite FL/DLBCL may share similar genotypic prognostic factors with *de novo* DLBCL.

Our study also suggests that patients with composite FL and DLBCL carrying the ABC subtype tended to have a shorter OS as compared to those with the GCB subtype, even though this finding was short of statistical significance and not incorporated into the CGI model. This is similar to the findings reported in Witte *et al*., 2018 where non-germinal centre derived composite FL and DLBCL had an inferior clinical outcome bordering on statistical significance as compared to cases with GCB subtype^26^. We compared these results to studies analysing the prognostic value of Han’s algorithm in *de novo* DLBCL. Older studies conducted in the pre-rituximab era generally suggest that the ABC subtype confers a poorer prognosis as compared to the GCB subtype in DLBCL^27,28^. However, newer studies incorporating data from patients treated with first-line chemotherapy of R-CHOP are divided on the prognostic utility of Han’s algorithm as a prognostic factor. Most studies report that Han’s algorithm has no prognostic value for DLBCL in the rituximab era^25,29^. One study compared patients treated with chemotherapy with and without rituximab and concluded that Han’s algorithm only held prognostic value in the group treated with chemotherapy alone^30^. Nevertheless, a few still report that the ABC subtype continues to be unfavourably associated with OS as compared to the GCB subtype, even as rituximab improves the OS for both subtypes^31^. In keeping with this data, our results in FL/DLBCL further suggest that Han’s algorithm probably has limited prognostic value in the rituximab era.

Interestingly, we also found that the presence of B-symptoms at diagnosis was an independent prognostic factor for composite FL/DLBCL and portends a poor OS. Together with the choice of first line chemotherapy regimen and eventual response to treatment, our findings highlight the relevance of clinical biomarkers even in the modern molecular genomic era.

Important limitations of our current study include a relatively small patient cohort derived from a single institution and its retrospective design. Additionally, we were not able to capture some of the data for individual immunohistochemical markers and genetic translocations. Nonetheless, our study is one of the largest to date in patients with composite histology of FL and DLBCL and incorporates clinical, immunohistochemical and molecular data.

In conclusion, 5 clinico-genotypic markers – presence of B-symptoms at diagnosis, non-CR to first-line chemotherapy, use of chemotherapy regimen other than R-CHOP, stage 3–4 lymphoma and presence of *MYC* and/or *BCL6* rearrangements all contribute to the generation of a prognostic index as independent prognostic factors for poor OS. This index should be further validated by future studies in composite FL and DLBCL.

## Supporting Information

Supporting Information.
