# Supplementary material for: A Clinico-Genotypic Prognostic Index for De Novo Composite Diffuse Large B-Cell Lymphoma Arising from Follicular Lymphoma in Asian patients treated in the Rituximab Era
Source: Sci Rep. 2020 Mar 9;10:4373. doi: 10.1038/s41598-020-61378-4 (PMC7062756; doi:10.1038/s41598-020-61378-4)
Supplement: Supplementary file 1 — Supporting Information. [file 41598_2020_61378_MOESM1_ESM.docx]

**A Clinico-Genotypic Prognostic Index for *De Novo* Composite Diffuse Large B-Cell Lymphoma Arising from Follicular Lymphoma in Asian patients treated in the Rituximab Era**

*Ryan Mao Heng LIM^1^*, Natalie Pei Xin CHAN*^1^*, Lay Poh KHOO*^2^*, Chee Leong CHENG^3^, Leonard TAN^3^, Eileen Yi Ling POON^2 4^, Nagavalli SOMASUNDARAM^2 4^, Mohamad FARID^2 4 5^, Tiffany Pooi Ling TANG^2 4 5^, Miriam TAO^2 4 5^, Soon Thye LIM^2 4 5 *^, Jason Yongsheng CHAN^2 4 5 6 *^

^1^Yong Loo Lin School of Medicine, National University of Singapore, Singapore

^2^Division of Medical Oncology, National Cancer Centre Singapore, Singapore.

^3^Department of Anatomical Pathology, Singapore General Hospital, Singapore

^4^SingHealth Duke-NUS Blood Cancer Centre, Singapore

^5^Duke-NUS Medical School, Singapore

^6^Cancer Science Institute of Singapore, National University of Singapore, Singapore

***Address for correspondence and reprint requests**:

Dr Jason Yongsheng CHAN

Division of Medical Oncology, National Cancer Centre Singapore, Singapore.

11 Hospital Drive, Singapore 169610

Tel: +65 6436 8000 Fax: 6227 2759 Email: Jason.chan.y.s@nccs.com.sg

Professor Soon Thye LIM

Division of Medical Oncology, National Cancer Centre Singapore, Singapore.

11 Hospital Drive, Singapore 169610

Tel: +65 6436 8000 Fax: 6227 2759 Email: lim.soon.thye@singhealth.com.sg

**Supplementary Tables and Figures**

**Supplementary Table 1a. Comparison of clinical and demographic characteristics between synchronous and metachronous FL/DLBCL**

| **Characteristic** | **Synchronous FL/DLBCL N (%)** | **Metachronous FL/DLBCL (N%)** | **P-value** |
| --- | --- | --- | --- |
| ***Total*** | 106 (100%) | 21 (100%) |  |
| ***Age (years)*** | | | |
| **>60** | 50 (47.2%) | 12 (57.1%) | 0.0564 |
| **<60** | 56 (52.8%) | 9 (42.9%) |  |
| ***Sex*** | | | |
| **Male** | 72 (67.9%) | 14 (66.7%) | 1.0000 |
| **Female** | 34 (32.1%) | 7 (33.3%) |  |
| ***Ethnicity*** | | | |
| **Chinese** | 71 (67.0%) | 16 (76.2%) | 0.5509 |
| **Malay** | 13 (12.3%) | 0 (0.0%) |  |
| **Indian** | 8 (7.5%) | 1 (4.8%) |  |
| **Others** | 14 (13.2%) | 4 (19.0%) |  |
| ***B-symptoms*** | | | |
| **Absent** | 74 (69.8%) | 17 (81.0%) | 0.4282 |
| **Present** | 32 (30.2%) | 4 (19.0%) |  |
| ***ECOG performance status*** | | | |
| **0** | 62 (58.5%) | 17 (81.0%) | 0.0828 |
| **1-4** | 44 (41.5%) | 4 (19.0%) |  |
| ***Stage*** | | | |
| **1-2** | 42 (39.6%) | 5 (23.8%) | 0.9101 |
| **3-4** | 64 (60.4%) | 16 (76.2%) |  |
| ***FLIPI*** | | | |
| **0-1** | 35 (33.0%) | 4 (19.0%) | 0.4346 |
| **2** | 23 (21.7%) | 6 (28.6%) |  |
| **3** | 48 (45.3%) | 11 (52.4%) |  |
| ***Response to chemotherapy*** | | | |
| **Complete response (CR)** | 71 (67.0%) | 8 (38.1%) | **0.0247** |
| **Non-CR** | 35 (33.0%) | 13 (61.9%) |  |
| ***First line chemotherapy regimen*** | | | |
| **R-CHOP** | 89 (84.0%) | 10 (52.4%) | **0.0007** |
| **Others*** | 17 (16.0%) | 11 (47.6%) |  |

Abbreviations: FLIPI, Follicular Lymphoma International Prognostic Index; IPI, International Prognostic Index

**Supplementary Table 1b. Comparison of histopathological and molecular characteristics between Synchronous and Metachronous FL/DLBCL**

|  | **Synchronous FL/DLBCL N (%)** | **Metachronous FL/DLBCL N (%)** | **P-value** |
| --- | --- | --- | --- |
| ***Number of lymph node involvement*** | | | |
| **1-3** | 81 (76.4%) | 12 (57.1%) | **<0.0001** |
| **≥ 4** | 25 (23.6%) | 9 (42.9%) |  |
| ***Serum LDH*** | | | |
| **Not elevated** | 35 (33.0%) | 5 (23.8%) | 0.4546 |
| **Elevated** | 71 (67.0%) | 16 (76.2%) |  |
| ***BCL2 expression*** | | | |
| **Negative** | 24 (24.2%) | 0 (0.0%) | **0.0121** |
| **Positive** | 75 (75.8%) | 20 (100.0%) |  |
| ***BCL6 expression*** | | | |
| **Negative** | 6 (7.0%) | 7 (38.8%) | **0.0014** |
| **Positive** | 80 (93.0%) | 11 (61.1%) |  |
| ***CD10 expression*** | | | |
| **Negative** | 32 (30.8%) | 6 (31.6%) | 1.0000 |
| **Positive** | 72 (69.2%) | 13 (68.4%) |  |
| ***MUM1 expression*** | | | |
| **Negative** | 24 (28.2%) | 9 (50.0%) | 0.0958 |
| **Positive** | 61 (71.8%) | 9 (50.0%) |  |
| ***Ki-67 expression*** | | | |
| **<90%** | 68 (72.3%) | 18 (94.7%) | 0.0403 |
| **>90%** | 26 (27.7%) | 1 (5.3%) |  |
| ***MYC expression*** | | | |
| **<40%** | 41 (70.7%) | 8 (72.7%) | 1.0000 |
| **>40%** | 17 (29.3%) | 3 (27.3%) |  |
| ***Cell of origin*** | | | |
| **ABC** | 62 (60.8%) | 9 (45.0%) | 0.2206 |
| **GCB** | 40 (39.2%) | 11 (55.0%) |  |
| ***BCL2 rearrangement*** | | | |
| **Negative/unknown** | 87 (82.1%) | 17 (81.0%) | 1.0000 |
| **Positive** | 19 (17.9%) | 4 (19.0%) |  |
| ***BCL6 rearrangement*** | | | |
| **Negative/unknown** | 90 (84.9%) | 20 (95.2%) | 0.3025 |
| **Positive** | 16 (15.1%) | 1 (4.8%) |  |
| ***MYC rearrangement*** | | | |
| **Negative/unknown** | 101 (95.3%) | 20 (95.2%) | 1.0000 |
| **Positive** | 5 (4.7%) | 1 (4.8%) |  |
| ***BCL6* and/or *MYC* rearrangement** | | | |
| **Negative/unknown** | 85 (80.2%) | 19 (90.5%) | 0.3613 |
| **Positive** | 21 (19.8%) | 2 (9.5%) |  |
| **Double-hit** |  | | |
| **Negative/unknown** | 103 (97.2%) | 20 (95.2%) | 0.5193 |
| **Positive** | 3 (2.8%) | 1 (4.8%) |  |

Abbreviations: LDH, lactate dehydrogenase; DLBCL, diffuse large B-cell lymphoma; ABC, activated B-cell-like; GCB, germinal centre B-cell-like


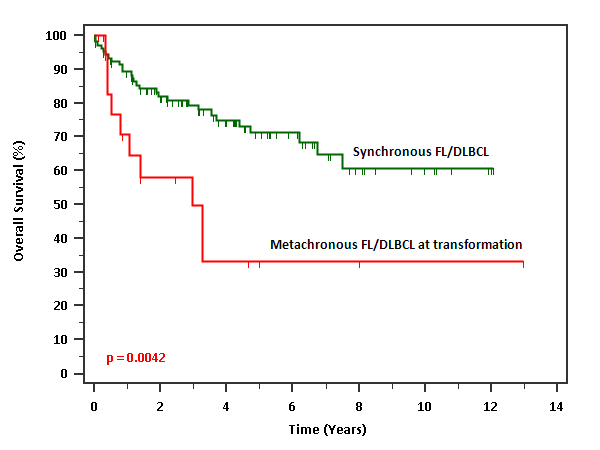


**Supplementary Figure 1. Survival outcomes of synchronous FL/DLBCL and metachronous FL/DLBCL at transformation.** Metachronous FL/DLBCL at transformation had a significantly worse OS as compared to synchronous FL/DLBCL (p = 0.0042).


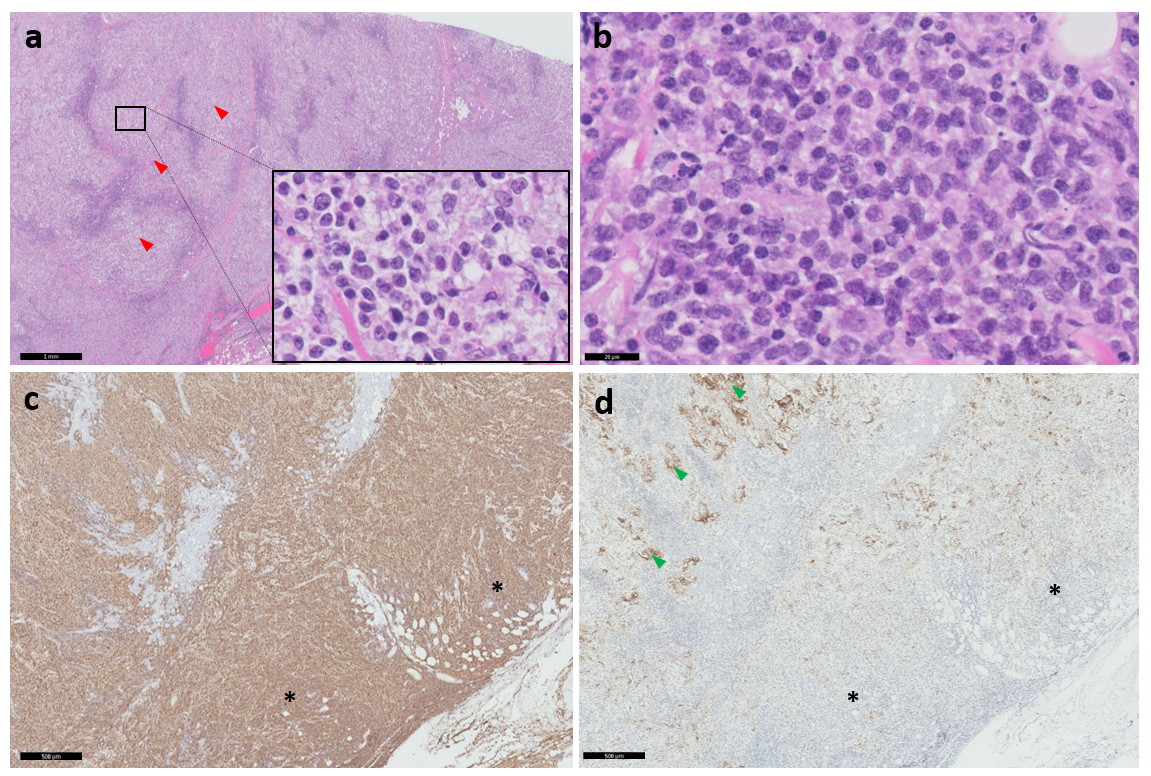


**Supplementary Figure 2. Representative histopathological images of composite FL/DLBCL.** (a) H&E stained section depicting a lymphoproliferative lesion that consists of areas with atypical follicular structures (red arrows). These correspond to similar foci with CD21 immunoreactive follicular dendritic meshworks (green arrows in sub-image (d) of this figure). These atypical follicular structures contain a mixed population of centrocytic and centroblastic cells (inset). In this example, the centroblasts predominate (> 15 per high-power field), commensurate with WHO grade 3A follicular lymphoma. (b) In the peripheries, the lesion consists of diffuse areas of large cells (c) staining positive for CD20 (asterisks), (d) while lacking recruitment of follicular dendritic meshworks as highlighted by immunoreactivity for CD21 (asterisks).
